# Seroprevalence of measles-specific IgG and genotype-specific neutralizing antibody responses in clinically confirmed cases during the 2021–2023 measles outbreaks in Liberia

**DOI:** 10.21203/rs.3.rs-9210123/v1

**Published:** 2026-04-14

**Authors:** Kalilu S. Donzo, Nicola Logan, TROY D. MOON, Ralph W. Jetoh, Margaret J. Hosie, Peter Kojo Quashie, Brian J. Willett, Kwadwo Asamoah Kusi

**Affiliations:** West African Centre for Cell Biology of Infectious Pathogens, University of Ghana, Legon-Accra, Ghana; Department of Biochemistry, Cell, and Molecular Biology, University of Ghana, Legon-Accra, Ghana; MRC-University of Glasgow Centre for Virus Research, Glasgow, Scotland; Tulane University School of Public Health and Tropical Medicine, New Orleans, Louisiana; National Public Health Institute of Liberia, Oldest Congo Town, Monrovia, Liberia; MRC-University of Glasgow Centre for Virus Research, Glasgow, Scotland; West African Centre for Cell Biology of Infectious Pathogens, University of Ghana, Legon-Accra, Ghana; MRC-University of Glasgow Centre for Virus Research, Glasgow, Scotland; Immunology Department, Noguchi Memorial Institute of Medical Research, College of Health Sciences, University of Ghana, Accra, Ghana

**Keywords:** Measles virus outbreak, MeV-specific IgG, neutralizing antibody, clinically-confirmed cases, seroprevalence, Liberia, neutralization breadth, measles containing vaccine, genotype–cross-neutralizing

## Abstract

**Background:**

Since 2021, Liberia has experienced disruptive outbreaks of measles across all of its 15 counties, despite the availability of a safe, effective vaccine. There is no data on the potential role of vaccine-induced immunity and no study on seroprevalence of measles-specific IgG in Liberia. Furthermore, there is no data on genotype-specific variation in antibody effectiveness in Liberia. This study evaluated the seroprevalence of measles virus (MeV)-specific IgG in clinically diagnosed patients in Liberia and assessed the breadth of neutralization against four genotypes.

**Methods:**

Samples collected retrospectively from 2021 to 2023 and prospective samples collected in 2023 from clinically diagnosed measles cases were analyzed in this study. Study participants included both vaccinated and unvaccinated individuals. MeV-specific IgG antibody was assessed using an enzyme-linked immunosorbent assay. Neutralizing antibodies against the Edmonston, B3, D4, and D8 MeV genotypes were measured using vesicular stomatitis virus pseudotype-based neutralization assays.

**Results:**

Overall, 69.9% of 199 individuals, including 100 vaccinees that were clinically diagnosed with measles, tested positive for MeV-specific IgG antibodies. MeV-specific IgG positivity was higher in vaccinated (52.5%) than in unvaccinated individuals (8.6%). Strong neutralization was observed against the D8 genotype in 98.9% of seropositive individuals, compared to 94.7% against Edmonston and D4 genotypes and 91.6% against B3. Significantly higher neutralization titers were observed in vaccinated compared to unvaccinated individuals for all four genotypes (p < 0.0001). There was a weak positive correlation between IgG levels, neutralizing titers, and participant age.

**Conclusion:**

The findings demonstrate strong genotype-cross-neutralizing immunity among vaccinated and seropositive patients, highlighting the critical role of measles vaccination in maintaining effective antibody protection. The significant percentage of seronegative patients indicates ongoing vulnerability to measles transmission and reinforces the need to improve vaccination coverage.

## BACKGROUND

Measles is a highly contagious airborne viral infection caused by measles virus (MeV) resulting in severe complications and sometime death. These severe complications are predominantly in young children due to immature immunity, waning maternal antibodies, and delayed vaccination (1). MeV is spread through respiratory droplets, and clinical symptoms typically include high fever, cough, conjunctivitis, and a characteristic maculopapular rash. Measles symptoms typically develop 10–14 days after exposure to the virus. The World Health Organization (WHO) case definition of measles categorizes a suspected case as a patient having a temperature over 38°C and a maculopapular rash. According to WHO, a clinical case of measles is defined as any patient presenting with a fever, a maculopapular rash, and at least one of the following symptoms: cough, coryza, or conjunctivitis.

Despite the availability of safe and effective vaccines, measles continues to pose a significant global health challenge. In 2023, over 107,500 measles-related mortalities occurred globally, largely among unvaccinated children under five years of age. In 2022, Measles Containing Vaccine dose 1 (MCV1) coverage declined to 83% globally, its lowest since 2008 (2). In Liberia, MCV1 coverage increased from 58% in 2021 to 82% in 2023, and MCV2 from 50% in 2019 to 60% in 2023 (3). However, Liberia has experienced a nationwide outbreak since 2021, with 14,192 confirmed cases and 98 deaths reported as of December 14, 2025 (4).

The B3 genotype remains the predominant endemic measles virus lineage circulating in sub-Saharan Africa, with sustained transmission documented across Western and Central Africa (5). This genotype has been repeatedly reported in Nigeria, Ghana, Cameroon, and The Gambia, reflecting its long-standing regional establishment (5). In contrast, the D4 and D8 genotypes show greater genetic heterogeneity and are more often associated with outbreaks and importation events, particularly in eastern and southern parts of the continent (6). D4 genotypes have been detected in countries such as Uganda, Tanzania, and South Africa, with many reports suggesting importation or short-term regional dissemination rather than persistent endemic circulation (6). Similarly, D8 genotype viruses have been identified in South Africa and Zimbabwe in recent surveillance years, although their occurrence has varied from year to year, indicating sporadic introductions rather than continuous transmission (6).

Serological surveillance is crucial for ascertaining immunity gaps and evaluating vaccination program performance. IgG seroprevalence surveys help assess population protection, while neutralization assays are crucial for measuring functional immunity, as Enzyme-Linked Immunosorbent Assay (ELISA) cannot differentiate neutralizing from non-neutralizing antibodies (4–5). Neutralizing antibodies targeting MeV hemagglutinin (H) and fusion (F) proteins are key to protection and reflect the antigenic variability of circulating genotypes (6, 7, 8). Previously, studies have shown genotype-specific differences in neutralizing responses. Lower titers against MeV genotype A compared with D4 and D8, reduced titers against genotype B3 in Iran, and variable responses across genotypes in the United Kingdom (UK) demonstrate the importance of assessing genotype-specific immunity (10–12, 34).

Liberia introduced MCV1 in 1978 and MCV2 in 2019, but there is no data on measles IgG seroprevalence, vaccine-induced immunity, or genotype-specific neutralization. This study aimed to assess MeV-specific IgG seroprevalence in clinically confirmed measles patients and to evaluate the neutralization breadth of MeV-specific IgG against four genotypes during Liberia’s ongoing outbreak.

## METHODS

### Study Design and Subject Selection Criteria

The study employed a cross-sectional design that consisted of two components: a retrospective evaluation using whole-blood samples obtained from individuals meeting the suspected case definition for MeV stored at the National Reference Laboratory of Liberia from December 2021 to August 2023 and a prospective study that collected samples from March 2023 to June 2023 of clinically diagnosed MeV patients at the Redemption Hospital in Monrovia, Liberia.

#### Separation of Plasma and Sera from Whole Blood.

We collected whole blood using BD Vacutainer^®^ cell preparation tubes, centrifuged for 30 minutes at 1800 × g at room temperature. Plasma was then transferred into 2 mL tubes and stored at − 80°C.

#### Enzyme-Linked Immunosorbent Assay (ELISA)

All samples were brought to 25°C, and MeV-specific IgG was quantified using a commercial ELISA kit (SERION ELISA classic measles virus IgG) according to the manufacturer’s instructions. The commercial ELISA kit uses recombinant MeV nucleoprotein derived from the Edmonston strain. Samples were diluted 1:100 (5 μL sample + 495 μL diluent), loaded with controls onto 96-well plates, and the plates were incubated for 60 minutes at 37°C. After the washing steps, plates were incubated for 30 minutes with anti-human IgG conjugate at 37°C and washed again. The substrate solution containing para-nitrophenylphosphate was added and incubated for 30 minutes at 37°C. The reaction was stopped using 1.2 N NaOH, and optical density was measured at 405 nm. Antibody titers (mIU/mL) were calculated using the manufacturer’s calibration specifications, with serostatus defined as < 150 (negative), 150–200 (equivocal), and > 200 (positive). A positive IgG result indicated prior infection or vaccination.

#### Cell Lines and Viruses

Human embryonic kidney (HEK) 293T cells were cultured and engineered to express the human SLAM1 receptor under standard culture conditions as described by Logan et al. SLAM1 (CD150), expressed on immune cells, is the primary receptor for wild-type MeV. Cells were cultured and maintained in DMEM supplemented with 10% FBS, 100 IU/mL penicillin-streptomycin, and 2 mM L-glutamine. For stable selection, HEK 293 SLAM1 cells were grown in medium containing 1 μg/mL puromycin and HEK 293T cells with 400 μg/mL G418. These conditions ensured consistent SLAM1 expression and reliable experimental performance. All reagents were provided by Hosie’s and Willett’s laboratories at the Centre for Virus Research, University of Glasgow.

#### Production of Vesicular Stomatitis Virus ΔG (VSV ΔG) MeV Pseudovirus

The VSV ΔG–MeV pseudoviruses were generated using four MeV genotypes (Edmonston, B3, D8, and D4). Plasmid vectors encoding each genotype’s H and F glycoproteins were synthesized, and pseudoviruses were produced by transfecting 2 × 10^6^ HEK 293T cells with 5 μg each of H and F plasmids. After 4 hours, cells were infected with VSV ΔG Luc (1.15 × 10^7^ TCID_50_; MOI 0.02) for 1 hour, then washed and incubated in 10 mL DMEM for 48 hours at 37°C/5% CO_2_. Supernatants were then filtered through a 0.45 μm filter, and viral titers were determined by TCID_50_ on HEK 293 SLAM1 cells.

#### Pseudotype-Based Neutralization Assay

The retrospective and prospective serum samples were serially diluted 4-fold from 1:16 to 1:32,768, and the diluted sera were incubated in triplicate with 2.5 × 10^3^ TCID_50_ of pseudotyped virus in 96-well plates for 1 hour at 37°C/5% CO_2_. Afterward, 2 × 10^4^ HEK 293 SLAM1 cells were added and incubated on plates for 48 hour before adding Steadylite Plus^™^ luciferase substrate and measuring luminescence on a Revvity EnSight^®^ plate reader.

### Data and Statistical Analysis

All data were analysed using GraphPad Prism 7.01 (GraphPad Software, San Diego, CA, USA) and R 4.3.1 (R Development Core Team, including ggplot2 for visualization). MeV-specific IgG levels were compared between vaccinated and unvaccinated groups using the two-tailed Mann–Whitney U test. Neutralizing antibody responses to the three wild-type genotypes (B3, D4, and D8) were evaluated in comparison with the vaccine genotype, Edmonston. A WHO reference serum product number NIBSC code 97/648 (3000 mIU/mL; protective level 125 mIU/mL) was diluted and included to define the protective threshold—the minimum measles-specific antibody level that confers protective immunity—in the pseudotype assay. Median neutralizing titers across genotypes were compared using the Kruskal–Wallis test and vaccinated versus unvaccinated titers were analyzed using the Wilcoxon matched-pairs signed rank test. Spearman’s rank correlation was used to assess the relationships between IgG status and age, as well as the associations between Edmonston, B3, D4, and D8 neutralization titers and age. Statistical significance was set at p-value < 0.05.

## RESULTS

### Demographics Characteristics

A total of one hundred and ninety-nine (199) samples were analysed for MeV-specific IgG (Table 1). Participant characteristics varied by vaccination status. Sex distribution did not differ significantly between groups (p-value = 0.50). There were statistically significant differences in age groups (p-value < 0.001): vaccinated participants were mainly 11–20 years old, while unvaccinated participants were also concentrated in this age range, and those with unknown status were mostly younger (< 5 years and 5–10 years). Sample type exhibited statistically significant differences (p-value < 0.001), with all unvaccinated participants drawn from prospective samples and all with unknown status from retrospective samples. Clinical presentations were similar, as all participants reported fever and maculopapular rash, with no differences between groups (fever: p-value = 1.0; rash: p-value = 1.0).

### Seroprevalence of Measles-Specific IgG

**Figure 1:**
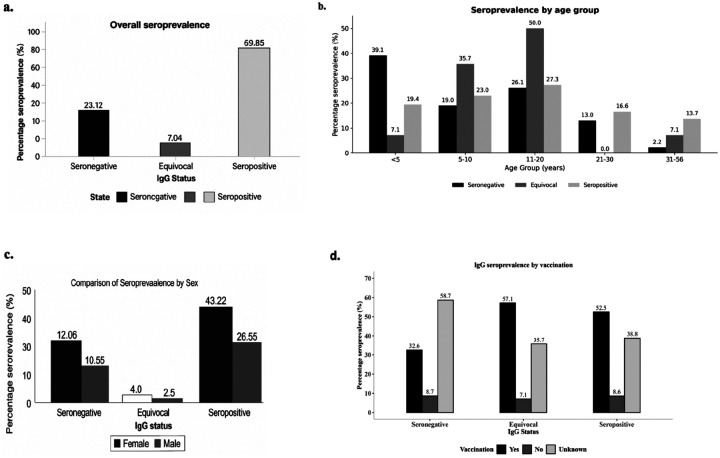
Comparison of measles-specific IgG seroprevalence. (a) overall seroprevalence among study participants, (b) seroprevalence across age groups, (c) seroprevalence by gender, and (d) seroprevalence based on vaccination history. All bar charts were generated using R version 4.3.1.

Out of 199 samples tested for MeV-specific IgG, 139 participants (69.9%) were IgG seropositive, with smaller proportions being seronegative (23.1%) or equivocal (7.0%) (Fig. 1a). The IgG status among participants varied by age group. Seronegativity was highest among children < 5 years of age (39.1%) with 19.4% being seropositive and 7.1% being equivocal. In the 5–10-year age group, seronegativity remained common (19.6%), while seropositivity increased to 23% and 37.5% were equivocal. Adolescents aged 11–20 years recorded the highest proportion of seropositive samples (27.3%), with 26.1% being seronegative, and 50% equivocal. Older adults aged 21–30 years showed lower seropositivity (16.6%) and no equivocal results, whereas those aged 31–56 years of age displayed a modest increase in seropositivity (13.7%) alongside low levels of seronegativity and equivocal findings (Fig. 1b). Gender differences were observed in seroprevalence of IgG (Fig. 1c). Female participants exhibited a higher proportion of seropositivity (43.2%) compared with male participants (26.6%), while sero-negativity and equivocal proportions were broadly similar between genders (Fig. 1c). Vaccination status was strongly correlated with being IgG seropositive (Fig. 1d). Vaccinees had the highest rate of seropositivity (52.5%) while unvaccinated participants demonstrated a decreased seropositivity rate (8.6%) (Fig. 1d).

**Figure 2:**
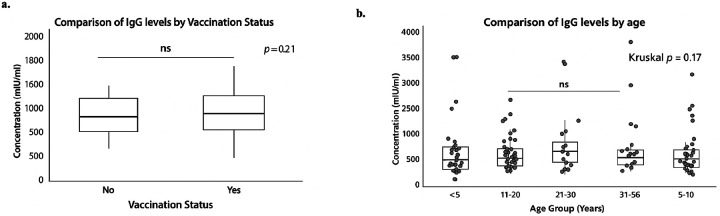
Comparison between levels of MeV IgG by vaccination status and age group. The Wilcoxon signed ranked test was used to compare the median between vaccinated vs unvaccinated and age group. A-B show the concentrations in Uml/ml of IgG antibody between vaccination status and age group. The bold lines reveal median values. A p-value less than 0.05 was deemed statistically significant.

MeV-specific IgG concentration levels did not differ significantly between vaccinated and unvaccinated individuals (p = 0.21; Fig. 2a). Similarly, no statistically significant differences in MeV-specific IgG levels were observed across age groups (> 5 years, 5–10 years, 11–20 years, 21–30 years, and 31–56 years; Fig. 2b). Although not statistically significant, there was a modest trend toward higher seropositivity with increasing age and vaccination status.

### Neutralizing Antibody Responses to Four MeV genotypes in Seropositive Individuals

**Figure 3:**
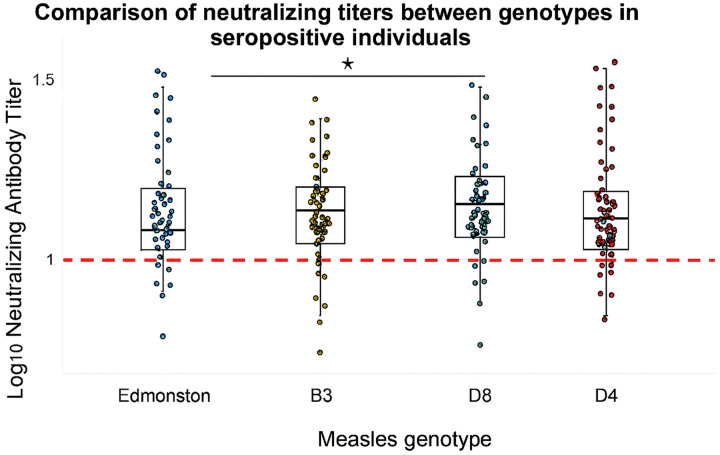
Comparison of MeV neutralization titers of seropositive individuals against four genotypes. The Kruskal–Wallis test was utilized to compare median values across the four groups. Bold lines denote the medians, and a p-value of less than 0.05 was considered statistically significant. The red dotted lines represent the reference titer defined by the third WHO standard serum (NIBSC 97/648).

The percentage of seropositive individuals with neutralizing antibody titers was determined for the four genotypes; our results revealed that 94.7% of seropositive individuals tested demonstrated neutralization titers against Edmonston, 91.6% against B3, 94.7% against D4, and 98.9% against D8 (Table 2). Also, we compared MeV-neutralizing antibody titres between three wild-type genotypes (B3, D4, and D8) and vaccine genotype, Edmonston. Our findings revealed that B3 and D8 have significantly higher titers than Edmonston, while D4 has the lowest median neutralizing titer, with more participants below the protective threshold (Fig. 3).

### Comparison of MeV neutralization titers with vaccination status

**Figure 4:**
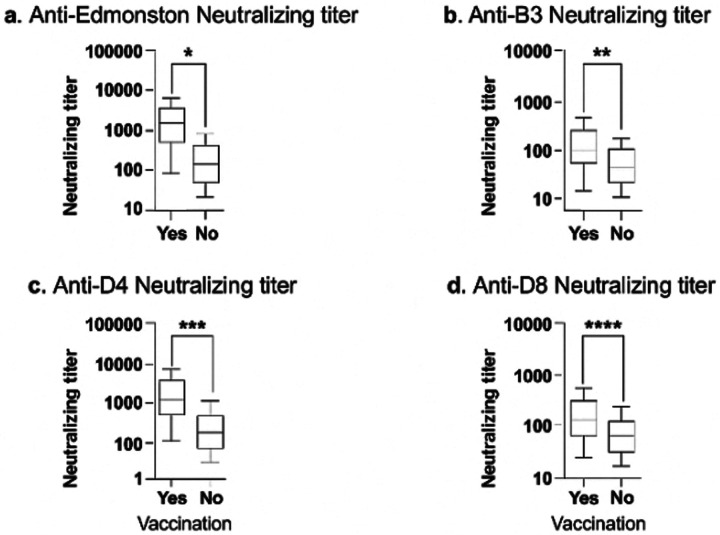
Comparison of neutralizing antibodies responses against Edmonston, B3, D4 and D8 by vaccinations status. The Wilcoxon signed ranked test was used to compare the median between the two groups. The bold lines reveal median values. p-value <0.05 was considered statistically significant. Statistical significance is indicated as follows: (a) Edmonston (* p-value = 0.0245), (b) B3 (** p-value = 0.0012), (c) D4 (*** p-value = 0.001) and (d) D8 (**** p-value < 0.001).

For all MeV genotypes assessed, vaccinated individuals displayed higher neutralization titers compared with the unvaccinated participants. This trend was the same for vaccine strain (Edmonston) as well as circulating wild-type B3, D4, and D8 genotypes tested (Fig. 4a–d).

### Correlation between Age and measles-specific IgG on Neutralizing Antibody Titers

**Figure 5:**
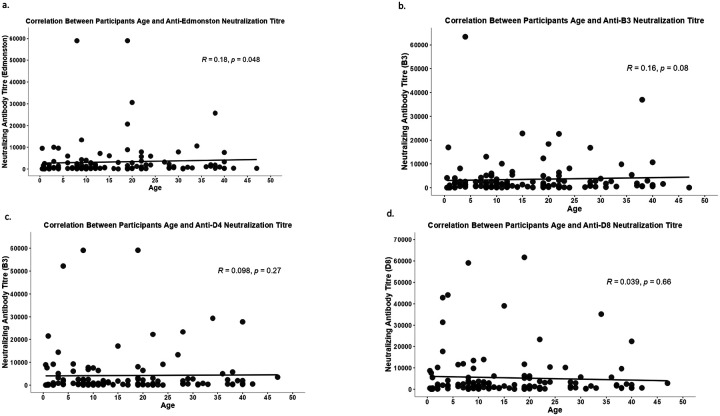
Correlation between neutralization titers (Edmonston, B3, D4, D8) and age. Spearman’s rank correlation rho was used to analyze the associations. p-value <0.05 was considered statistically significant.

**Figure 6:**
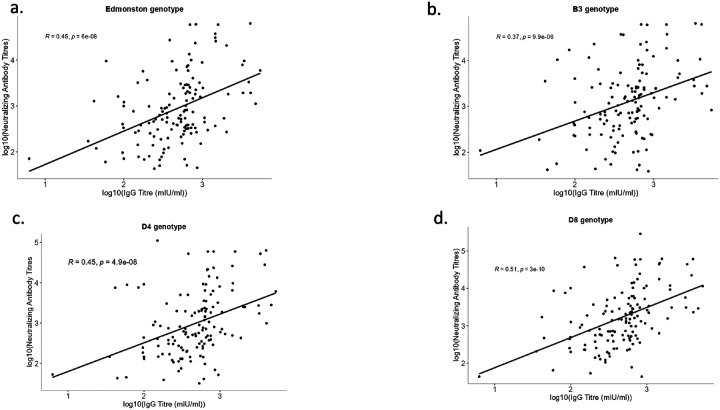
Correlation between measles-specific IgG and neutralization titers against (a)Edmonston (b) B3 (c) D4 (d) D8. Spearman’s rank correlation rho was used to analyze the associations. p-value <0.05 was considered statistically significant.

Neutralization titers against the Edmonston genotype showed a weak but statistically significant positive correlation with increasing age (Fig. 5a). Similarly, neutralization titers against the B3 and D4 genotypes showed a weakly positive correlation with increasing age, though neither was statistically significant (Figs. 5b and 5c). Since not all IgG antibodies neutralize, and low IgG still corresponds to neutralizing activity, we subsequently assessed the correlation between measles-specific IgG concentrations and neutralization titers. There was a moderate positive correlation between neutralization titers against D4, Edmonston, and measles IgG antibodies that was strongly statistically significant (Figs. 6a and 6c). The correlation between neutralization titers against D8, B3, and measles IgG antibodies ranged from moderate to strong and weak to moderate positive correlation, respectively (Figs. 6b and 6d).

## DISCUSSION

Seroprevalence studies offer critical understanding of the transmission patterns and population-level impact of infectious diseases. By measuring pathogen-specific antibodies, they can help in identifying previous exposure, estimate progress toward herd immunity thresholds, and guide public health interventions. Neutralizing antibody titers, in particular, are widely considered robust correlates of protective immunity, with higher titers generally associated with reduced risks of reinfection and severe disease. Although genotypes responsible for the current outbreak have not been identified, B3 was believed to be circulating in the West African region, including Liberia, in 2010 (5).

Previous studies have shown substantial differences in the seroprevalence of measles IgG in different settings. Zahoor et al. (8) and Adekola et al. (9) reported seroprevalence around 73.48% and 29.2% while Inuwa et al. (10) and Smetana et al. (11) found levels of seroprevalence of 61.6% and 70%. In contrast, significantly higher seroprevalences of measles IgG, ranging from 92% to 99%, were reported in the DRC (12), other African settings (13), China (14) and Vietnam (15). These variances likely reflect differences in national vaccination efficacy, historical patterns of measles transmission, and the role of natural infection in population immunity (16). The present study found an overall seroprevalence of 69.9%, with a seropositivity rate of just 52.5% among vaccinated individuals, suggesting immunity gaps in Liberia. The relatively low coverage of MCV1 and particularly MCV2 in Liberia could be contributing to these gaps, underscoring the need for strengthened routine vaccination and targeted strategies. Further study is necessary to understand the underlying factors contributing to low immunity, despite an MCV1 coverage of roughly 82%.

Naturally acquired immunity also affects seroprevalence patterns. In some regions, unvaccinated individuals, especially those aged 30 and above, have seroprevalence that sometimes exceeds 95%, mostly attributable to past encounters with MeV (13). However, some studies like Manirakiza et al. (17) showed a decline in measles immunity among specific populations despite vaccination programs, highlighting continued susceptibility in some demographic groups. In contrast to these studies, the present study found significant age-related differences in seropositivity with decreased immunity in children under 5, young adults (21–30 years), and adults (31–56 years). Decreased seropositivity in young infants may indicate limited vaccination coverage or waning of maternal antibodies. Stronger or higher titers among school-aged children and teenagers suggest stronger vaccine-derived protection in these demographics as compared to younger children and adults. Together, these findings highlight a changing landscape of susceptibility to measles, with increasing vulnerability in infants and older adults. Age-specific differences influence epidemic risk, especially in areas with low vaccination rates or late second-dose administration. Enhancing regular vaccination, improving maternal immunity, and bolstering catch-up campaigns for adolescents and young adults may be critical for attaining and maintaining measles eradication goals.

Earlier studies have consistently demonstrated waning neutralizing antibodies post-MMR vaccination, prompting concerns over long-term protection. An observational study of adolescents and young adults found that only 23.2% of individuals vaccinated with two doses of MMR maintained antibody levels above the protective threshold 7.4 years later, compared to 8.6% seropositivity among unvaccinated individuals (19). Modelling-based analyses have indicated annual declines of 9.7% in neutralizing antibodies following the first dosage of MMR and 4.8% after the second dose of MMR (20). Data from the United States similarly found a seronegativity rate of 33% two decades after MMR vaccination (21). These findings are consistent with the present study’s findings and collectively highlight an increasing risk among vaccinated populations and indicate a necessity to more accurately identify individuals who have titers below protective levels. The declines in neutralizing titers observed in earlier studies align with the present study’s finding. Decreased protective titers seen in individuals may suggest limited exposure or suboptimal immune responses to past vaccinations (18). These data collectively emphasize the need for enhanced monitoring of post-vaccination immunity and more study to elucidate the drivers and clinical consequences of declining measles antibodies.

Sero-epidemiological studies have also indicated considerable geographical variation in measles susceptibility. For example, a serosurvey in Romania has revealed a measles seronegativity of 23% (22), while a cross-sectional survey in Oman identified an even greater susceptibility among individuals aged 15–20 years (23). In contrast, lower seronegativity (12.3%) among vaccinees has been reported in Iran (24). Ristić et al. has also found a susceptibility threshold above WHO’s recommended susceptibility threshold of ≤ 5%. In the Central African Republic, only 51.3% and 27.6% had detectable measles IgG among vaccinees and unvaccinated individuals, respectively (17). The seronegativity rate of 23.12% found in the present study among participants aligns with these data, suggesting that approximately 1 in 4 participants lacked protective antibodies. Particularly concerning was the low seropositivity (52.5%) among vaccinated participants, despite Liberia’s reported MCV1 coverage of approximately 82%.

Sex-related differences in vaccine-induced immune responses have been reported. Females tend to generate larger measles-specific antibody titers post-vaccination, whereas males may have a more rapid reduction in these titers (25). Evidence from various settings corroborates these trends. For instance, studies from India (26), Europe (27) and the US (14) agreed with these trends. The present study found higher seropositivity among females (43.2%) than males (26.5%) consistent with this pattern. Additionally, across-sectional study of children above 15 years who received the MMR vaccine at 12–15 months indicated significantly higher prevalence of measles-specific IgG antibodies in females than in males (28). A regression analyses identified age at vaccination and female sex as the two principal determinants of long-term antibody persistence following MMR vaccination (29). These demographic differences are epidemiologically crucial for maintaining long-term protection against measles. The lower seropositivity seen in men may result in small yet epidemiologically important zones of susceptibility that might facilitate continued viral transmission. The high transmissibility of measles exacerbates the risk of outbreaks due to immunization gaps. To maintain herd immunity and avert a recurrence of measles, it is imperative to implement improved monitoring of male immunity and to conduct targeted vaccinations as necessary.

This study showed that participants mounted strong neutralizing responses against all four tested MeV genotypes. Titers were significantly higher for B3 as compared to Edmonston, suggesting that wild-type viruses may elicit more robust humoral responses than the attenuated vaccine lineage. Genotype D8 also produced higher titers than D4, indicating genotype-specific differences despite overall antigenic conservation. These findings align with reports of broad cross-neutralization among Edmonston, D4, and D8 (30), though variability has been noted, such as lower B3 titers in vaccinated individuals in Iran compared with H1, D4, and A genotypes (31). Further work is needed to understand molecular and structural drivers underlying the stronger responses observed for B3 and D8.

### Conclusions

The findings demonstrate strong genotype–cross neutralizing immunity among vaccinated and seropositive patients, highlighting the critical role of measles vaccination in maintaining effective antibody protection. However, the higher titers against wild-type strains compared to the vaccine strain raises concerns regarding the long-term durability of vaccine-induced immunity. The significant percentage of seronegative patients in our study indicates ongoing vulnerability to measles transmission and reinforces the need to improve vaccination coverage.

## Figures and Tables

**Table 1:** Demographic, and clinical characteristics of participants.

*Characteristics*	N	Vaccinated N = 100^1^	Unvaccinated N = 17^1^	Unknown N = 82^1^	p-value^2^
Sex	199				0.5
Female		62 (62.0%)	8 (47.1%)	48 (58.5%)	
Male		38 (38.0%)	9 (52.9%)	34 (41.5%)	
Age Group	199				<0.001
< 5 yrs		8 (8.0%)	4 (23.5%)	34 (41.5%)	
5–10 yrs		21 (21.0%)	2 (11.8%)	23 (28.0%)	
11–20 yrs		34 (34.0%)	8 (47.1%)	15 (18.3%)	
21–30 yrs		19 (19.0%)	3 (17.6%)	7 (8.5%)	
31–56 yrs		18 (18.0%)	0 (0.0%)	3 (3.7%)	
Sample Type	199				<0.001
Prospective		86 (86.0%)	17 (100.0%)	0 (0.0%)	
Retrospective		14 (14.0%)	0 (0.0%)	82 (100.0%)	
Fever	199				1.0
Yes		100 (100.0%)	17 (100.0%)	82 (100.0%)	
Maculopapular Rash	199				1.0
Yes		100 (100.0%)	17 (100.0%)	82 (100.0%)	
Cough	199				0.11
Yes		97 (97.0%)	17 (100.0%)	74 (90.2%)	
No		3 (3.0%)	0 (0.0%)	8 (9.8%)	
Conjunctivitis	199				0.2
Yes		37 (37.0%)	3 (17.6%)	33 (40.2%)	
No		63 (63.0%)	14 (82.4%)	49 (59.8%)	

1 n (%)

2 Pearson’s Chi-squared test; Fisher’s exact test

**Table 2:** The percentage of seropositive individuals with neutralizing titers exceeding the protective threshold.

Genotype	Percentage Protected (%)
Edmonston	94.7
B3	91.6
D4	94.7
D8	98.9

## Data Availability

All data supporting this study conclusion are all included within the article. Additional datasets are available from the corresponding author upon request.

## Abbreviations

DRC: Democratic Republic of Congo
DMEM: Dulbecco’s Modified Eagle Medium
EIA: Enzyme Immuno-assay
ELISA: Enzyme-Linked Immuno Assay
FBS: Fetal Bovine Serum
HEK: Human embryonic kidney
HRP: Horseradish peroxidase
IgG: Immunoglobulin G
IgM: Immunoglobulin M
IRB: Institutional Review Board
MCV: Measles Containing Vaccine
MCV1: Measles Containing Vaccine 1
MCV2: Measles Containing Vaccine 2
MeV: Measles Virus
MOI: Multiplicity of Infection
MRC: Medical Research Council
NPHIL: National Public Health Institute of Liberia
NPHRL: National Public Health Reference Laboratory
OD: Optic density
PNA: Pseudovirus Neutralization Assay
PREVSL: Partnership for Research in Emerging Viral Infections Sierra Leone
PRNT: Plaque Reduction Neutralization Test
SLAM: Signaling Lymphocytic Activation Molecule
UK: United Kingdom
VSVΔG: Vesicular Stomatitis Virus Delta G
WACCBIP: West African Centre for Cell Biology of Infectious Pathogens
WHO: World Health Organization
